# Therapeutic Effect of *Abelmoschus manihot* on Type 2 Diabetic Nonproliferative Retinopathy and the Involvement of VEGF

**DOI:** 10.1155/2020/5204917

**Published:** 2020-04-30

**Authors:** Yue Zhao, Xu Yu, Yan Lou, Xinyi Sun, Boyu Zhu, Weilong Xu, Lei Zhou, Hao Wu, Qingzi Jin, Heng Wang, Jianjiang Shen, Jiangyi Yu, Xiaofei An

**Affiliations:** ^1^Department of Endocrinology, Affiliated Hospital of Nanjing University of Chinese Medicine, Nanjing, China; ^2^Department of Ophthalmology, Affiliated Hospital of Nanjing University of Chinese Medicine, Nanjing, China; ^3^Department of Laboratory, Affiliated Hospital of Nanjing University of Chinese Medicine, Nanjing, China

## Abstract

**Objective:**

To evaluate the efficacy of *Abelmoschus manihot* in treating type 2 diabetic nonproliferative retinopathy.

**Methods:**

It was a randomized controlled clinical trial. The recruited eighty subjects with type 2 diabetic nonproliferative retinopathy were randomly divided into treatment group and control group. The two groups received basic treatments including control of blood glucose, blood pressure and blood lipid, management of diet, exercise and health education, and monitoring of relevant indicators. Additionally, the treatment group was given oral administration of *Abelmoschus manihot*. All subjects were followed up on monthly basis for consecutive six months. The related parameters including diabetic retinopathy (DR) incidence rates, “Early Treatment Diabetic Retinopathy Study” (ETDRS) vision scores, retinal thicknesses in macular region, serum vascular endothelial growth factor (VEGF) levels, and biochemical indicators of both groups before and after treatment were accurately collected and statistically analyzed.

**Results:**

There were no significant differences of DR severity levels, ETDRS vision scores, macular retinal thicknesses such as cube average thickness (CAT), central subfield thickness (CST), and cube volume (CV), and serum VEGF levels between two groups before treatment. Meanwhile, there were no significant differences of demographic characteristics, case terminations, blood glucose, blood lipid, blood pressure, biochemical indicators of hepatorenal function, hypoglycemic drugs, hypotensive drugs, and other basic treatments between two groups during six months treatment. The present study suggested that the remission rate of DR and the ETDRS vision score in the treatment group were significantly higher than those of the control group (remission rate: 25.4% vs 9.3%, *P*=0.01; ETDRS score: 78 (72, 82) vs 72 (67, 80), *P*=0.0002) while the progression rate of DR in the treatment group was significantly lower than that of the control group (progression rate: 4.2% vs 18.7%, *P*=0.007) after six months treatment. In addition, the CAT, CST, CV, and serum VEGF levels of the treatment group were significantly improved after the treatment (CAT: 286 (278, 302) vs 282 (270, 295) *μ*m, *P* < 0.0001; CST: 251 (239, 274) vs 248 (235, 265) *μ*m, *P* < 0.0001; CV: 10.3 (10.0, 10.9) vs 10.1 (9.7, 10.6) mm^3^, *P* < 0.0001; VEGF: 0.21 (0.14, 0.58) vs 0.16 (0.10, 0.23) ng/ml, *P*=0.0026), while there were no significant differences of the control group before and after treatment (CAT: 287 (279, 294) vs 287 (279, 295) *μ*m, *P*=0.27; CST: 250 (240, 266) vs 252 (238, 266) *μ*m, *P*=0.72; CV: 10.4 (10.1, 10.6) vs 10.4 (10.1, 10.7) mm^3^, *P*=0.53; VEGF: 0.21 (0.13, 0.66) vs 0.23 (0.12, 0.64) ng/ml, *P*=0.85).

**Conclusion:**

The study offered the novel evidence for the therapeutic effect of *Abelmoschus manihot* on type 2 diabetic nonproliferative retinopathy, which was associated with improved VEGF. This trial is registered with ChiCTR1800019292.

## 1. Introduction

As one of the major microvascular complications of diabetes, diabetic retinopathy (DR) is regarded as the leading cause of vision loss in middle-aged and elderly people all over the world [1]. A meta-analysis including 35 studies and 22,896 diabetes patients showed that the incidence of DR in diabetes was about 34.6%, among which the proliferative diabetic retinopathy (PDR) took up 6.96%, the diabetic macular edema (DME) about 6.81%, and the vision-threatening diabetic retinopathy (VTDR) about 10.2% [2]. With the extension of diabetic duration, the prevalence of DR keeps increasing [3] and the global medical cost required for the prevention and treatment of DR is also rising [1]. DR is becoming increasingly harmful to human health and burdened to the medical care system. Therefore, it is very meaningful to explore effective agents or treatment for preventing the progression of DR.

According to Diabetic Retinopathy Preferred Practice Pattern Guideline (Version 2017) issued by American Academy of Ophthalmology, strict control of blood glucose, blood pressure, blood lipid, and other metabolic disorders is taken as the main method of secondary prevention of DR. The antivascular endothelial growth factor (VEGF) therapy, panretinal photocoagulation, and vitrectomy could be adopted when severe nonproliferative diabetic retinopathy (NPDR), PDR, or clinically significant macular edema (CSME) occurs [4]. Several studies indicated that intravitreal dexamethasone (DEX) implant was effective for long term and safe to DME and diabetic tractional retinal detachment [5–8]. Additionally, the improvement of vision and macular retinal thickness by DEX was in both eyes that were treatment-naive and eyes refractory to anti-VEGF treatment [9]. However, all these surgical treatments are required to be operated by experienced ophthalmologists. For endocrinologists and general physicians who are responsible for DR management, it is essential to select the appropriate methods to prevent further progression at the early stage of DR. Although the first-line treatment including the control of blood glucose, blood pressure, and blood lipids has been adopted in current clinical practice, the development of DR still remains not to be mitigated and irreversible.

The recent study indicated the validity of the commonly used anti-DR drugs such as alprostadil, antioxidants, antithrombotics, and protein kinase-C inhibitors was lack of evidence from large-sample clinical research [10]. It seems that searching for some complementary and alternative agents from traditional Chinese medicine for DR may partly alleviate this dilemma. The studies showed that some herbal medicines reduced the apoptosis of retinal pericytes (RPCs), maintained the function of retinal endothelial cells (RECs), and inhibited retinal neovascularization by improving the oxidative stress and inflammatory state of DR [11]. The present six-month prospective clinical study was performed to investigate the clinical efficacy and possible mechanism of *Abelmoschus manihot*, a traditional Chinese medicine in treating type 2 diabetic nonproliferative retinopathy [12].

## 2. Methods

### 2.1. Study Design

Eighty eligible type 2 diabetic patients with nonproliferative retinopathy who met the following criteria were recruited in this randomized controlled clinical trial. Inclusion criteria include the following: (1) type 2 diabetes with nonproliferative retinopathy diagnosed by fundus photography or fluorescein angiography; (2) age 18∼70 years old, male or female; (3) in the last 12 weeks, the highest blood glucose <16.7 mmol/L, blood pressure ≤140/90 mmHg, and body mass index (BMI) <30 kg/m^2^; (4) completely understanding and signing informed consent. Exclusion criteria were as follows: (1) acute diabetic complications in last 4 weeks such as diabetic ketoacidosis or diabetic nonketogenic hyperosmolar syndrome; (2) serious chronic diabetic complications such as diabetic nephropathy with nephrotic syndrome, severe diabetic peripheral neuropathy, or diabetic lower-extremity artery occlusion; (3) retinal vascular occlusion, uveitis, age-related macular degeneration, hypertensive retinopathy, or other diseases leading to macular edema; (4) severe cataract affecting vision test or glaucoma limiting mydriasis; (5) serious systemic diseases such as cancer, severe inflammation, AIDS, heart failure, end-stage renal disease, respiratory failure, and hematologic or mental disorder; (6) pregnant or lactating women, allergic constitution, or poor adherence.

The randomized method in this study was block randomization with 4 subjects in one block and 20 blocks in total. The random arrangement (random coding table) for 80 subjects was generated by SAS statistical software. All subjects were assigned to a number from 1 to 80 according to the chronological order of enrollment and then randomly divided into the treatment group and control group, each with 40 cases.

All subjects were enrolled to receive comprehensive medical history collection, physical examination, test of blood glucose, blood lipid and other biochemical indicators, fundus photography, “Early Treatment Diabetic Retinopathy Study” (ETDRS) vision test, optical coherence tomography (OCT) examination, and serum VEGF assay. Both groups were given basic treatments including control of blood glucose, blood pressure and blood lipid, management of diet, exercise and health education, and monitoring of relevant indicators according to “China Guideline for Type 2 Diabetes.” The treatment group was additionally given oral administration of *Abelmoschus manihot* (Jiahua tablets) with 1.8 grams per time and three times every day. Jiahua tablets, as the semiextract tablets of *Abelmoschus manihot*, were produced by the Affiliated Hospital of Nanjing University of Chinese Medicine with production approval code Z04000511.

**Figure 1:**
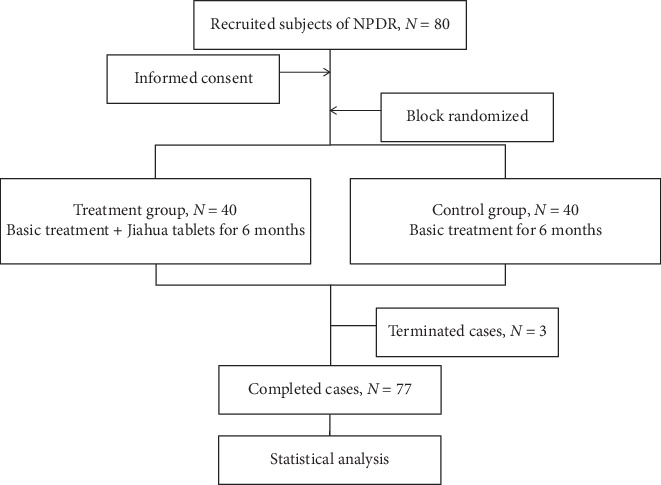
Study design and procedure.

All subjects were followed up on monthly basis for consecutive six months. The related indicators including DR incidence rates, ETDRS vision scores, macular retinal thicknesses, and serum VEGF levels of both groups before and after treatment were tested and compared to evaluate the efficacy of Jiahua tablets in treating DR. The research procedure was shown in Figure 1.

### 2.2. Main Observation Items and Testing Instruments

The NPDR severity levels (mild/moderate/severe) were assessed by the fundus camera (Kowa Nonmyd 7). The vision scores were evaluated by using the ETDRS vision chart. The retinal thicknesses in macular region were measured by the OCT instrument (Cirrus HD-OCT 5000, Carl Zeiss Meditec). The serum VEGF levels were assayed with the human VEGF-A ELISA kit (NJJCBIO Company, Nanjing, China) by the ELISA microplate reader (Bio Tek ELx800). The biochemical indicators including fasting blood glucose (FBG), postprandial blood glucose (PBG), glycosylated hemoglobin A1c (HbA1c), total cholesterol (TC), triglyceride (TG), high-density lipoprotein cholesterol (HDL), low-density lipoprotein cholesterol (LDL), alanine aminotransferase (ALT), aspartate aminotransferase (AST), blood urea nitrogen (BUN), serum creatinine (Scr), and estimated glomerular filtration rate (eGFR) were tested by the automatic chemistry analyzer (Beckman Coulter AU5800). The demographic characteristics and vital signs were also collected.

### 2.3. Relevant Attentions in the Study

When an adverse reaction of some subject was detected, the chief researcher needed to decide how to conduct further treatment and whether to discontinue the study of this subject. When a severe adverse event occurred, the research leader should immediately take necessary actions to protect the safety of the subject and, meanwhile, fill out the “severe adverse events report form” and report to the ethics committee of research institute within 24 hours to ensure that the reporting process meets all legal and regulatory requirements.

Part of subjects was considered to be terminated cases for some reasons as follows. Severe adverse events or other adverse reactions occurred; the study plan was obviously deviated (e.g., poor compliance of medication), and researchers thought it difficult to evaluate drugs' effect; subjects did not want to continue and applied for withdrawing from the study.

As for quality control and data management, all researchers needed to pass good clinical practice training and strictly follow the trial protocol in the study. Meanwhile, relevant laboratory technicians should comply with the standard operation procedure (SOP) of index testing, and the testing results should be traceable. The repetitive tests of fundus photography, ETDRS vision, OCT levels, and other indicators were performed by the same operators with same instruments according to the SOP. All subjects' data collected in the study were recorded in case report forms by researchers according to the SOP and checked regularly by quality controllers to ensure the authenticity, timeliness, accuracy, and integrity.

The enrolled subjects were randomly divided into the treatment group and control group according to the random coding table. All patients' indicators including biochemical indicators, blood pressure, DR severity, ETDRS score, macular retinal thickness, and serum VEGF were tested by special assessors of this study. However, the assessors were masked to grouping details. The intraclass correlation coefficients (ICC) of these indicators were calculated with the two-way random model and single absolute agreement type by SPSS 22.0. The estimated ICC values ranged from 0.85∼0.92. The interobserver reliability and test-retest reliability of indicators were considered to be valid according to the statistical principle.

### 2.4. Ethics

This trial was approved by the Ethics Committee of Affiliated Hospital of Nanjing University of Chinese Medicine (approval number: 2018NL-104-03) and registered the Chinese Clinical Trial Registry (registration number: ChiCTR1800019292). All subjects were enrolled in this study with signed informed consents which explained in detail the study's objective and procedure, possible benefits and risks, rights, and obligations.

### 2.5. Statistical Analysis

Values conforming to normal distribution were expressed as mean ± standard deviation ($\bar{x}$ ± SD), while those not conforming to normal distribution were expressed as median with interquartile range (M(Q1, Q3)). The measurement data were analyzed by *t* test (paired *t* test) or rank-sum test (paired rank-sum test) according to whether they conformed to the normal distribution, while the categorical data were mainly analyzed by chi-squared test or Fisher's exact test. It was two-sided test, and “*P* ≤ 0.05” was considered to be statistically significant. The statistic software was SAS 9.4 (SAS Institute Inc, Cary, North Carolina).

## 3. Results

**Figure 2:**
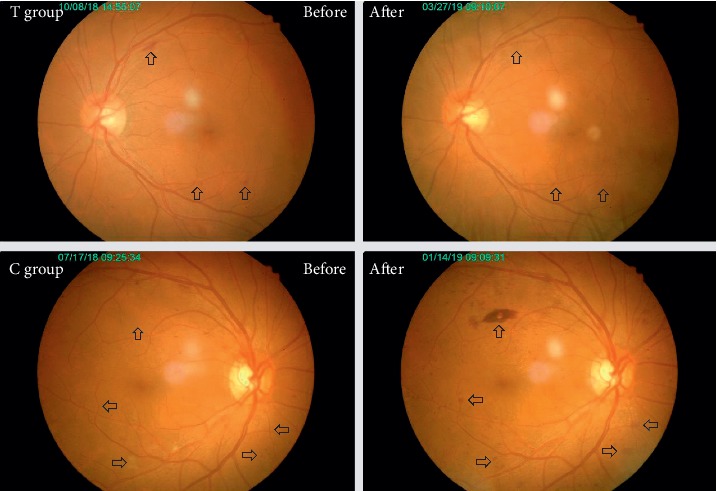
The typical changes of fundus photographs before and after treatment in the two groups. As indicated by the arrows in the figure, the retinal hemorrhage and exudation in the treatment group were significantly reduced after six months' treatment. In contrast, the retinal hemorrhage and exudation in the control group were significantly increased. T: treatment; C: control.

**Figure 3:**
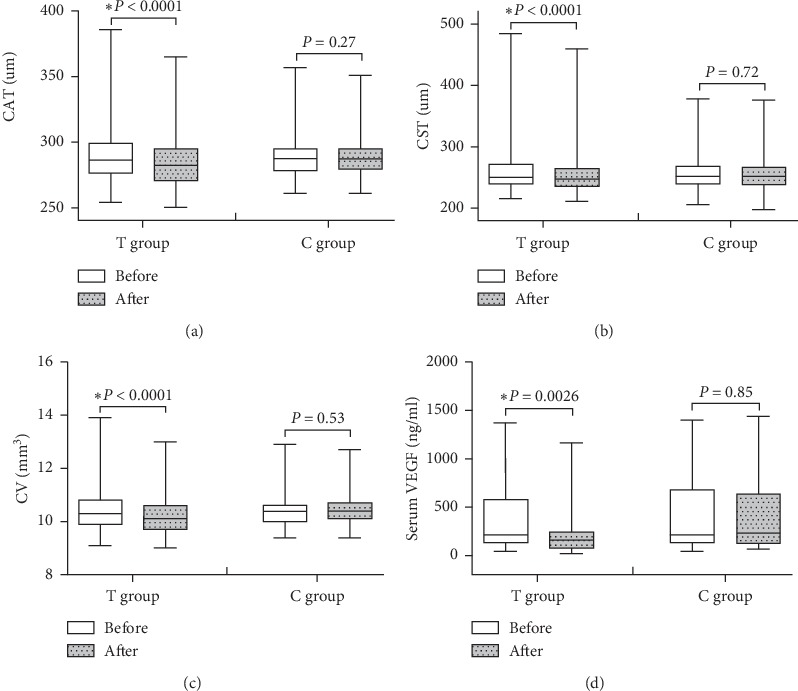
Comparisons of CAT, CST, CV, and VEGF of two groups before and after treatment. The treatment group's CAT, CST, and CV levels (a, b, and c) were all significantly lower after treatment, and there were no significant differences of control group's CAT, CST, and CV levels before and after treatment. The serum VEGF level (d) in the treatment group was significantly lower after treatment, and there was no significant difference of control group's VEGF before and after treatment. T: treatment; C: control.

**Figure 4:**
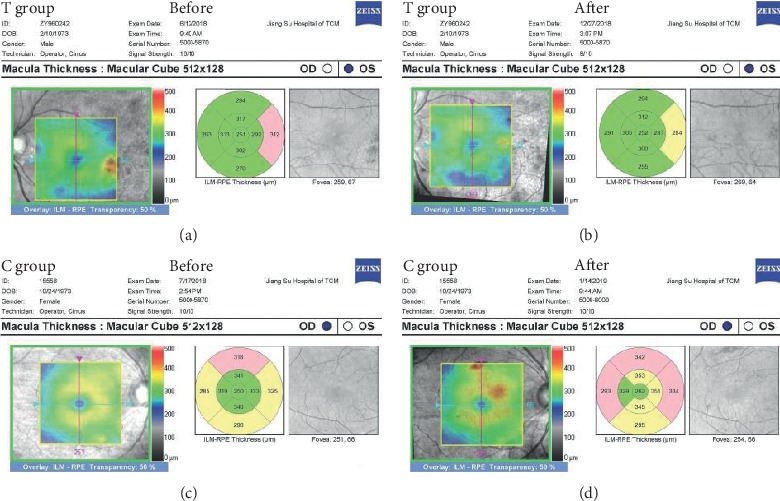
The typical changes of OCT images and parameters before and after treatment in the two groups. As shown in the figure, the macular thicknesses in the treatment group were significantly lower after six months' treatment. In contrast, the macular thicknesses in the control group were not significantly reduced. T: treatment; C: control.

1. As shown in Table 1, there were no significant differences of demographic characteristics including sex, age, DM duration, and BMI between the two groups.
2. Three cases subjects were terminated during the six-month treatment period including one case with automatic quit, one case with poor compliance in the treatment group, and another case with automatic quit in the control group. There were no significant differences in case terminations between the two groups as shown in Table 2.
3. There were no significant differences of blood glucose, blood lipid, blood pressure, and biochemical indicators of liver and kidney function between two groups before and after treatment as shown in Table 3.
4. Before treatment, eight unilateral eyes were excluded for observation including four with severe cataract and one with blindness due to trauma in the treatment group and two with severe cataract and one with severe nondiabetic macular disease in the control group. After six months' treatment, two cases (4 eyes) in the treatment group and one case (2 eyes) in the control group were terminated and excluded for observation.
5. There were 31 eyes with mild NPDR, 31 eyes with moderate NPDR, and 13 eyes with severe NPDR in the treatment group, while 31 eyes with mild NPDR, 36 eyes with moderate NPDR and 10 eyes with severe NPDR in the control group before treatment. There was no significant difference of NPDR severity between two groups before treatment as shown in Table 4.
6. However, after six months' treatment, there were 42 eyes with mild NPDR, 25 eyes with moderate NPDR, and 4 eyes with severe NPDR in the treatment group, while 27 eyes with mild NPDR, 35 eyes with moderate NPDR, and 13 eyes with severe NPDR in the control group. There was a significant difference of NPDR severity between two groups after treatment (*P*=0.008) as shown in Table 4.
7. Additionally, the remission rate of NPDR severity in the treatment group was significantly higher than that of the control group (25.4% vs 9.3%, *P*=0.01) and the progression rate of NPDR severity in the treatment group was significantly lower than that of the control group (4.2% vs 18.7%, *P*=0.007) as shown in Table 5. The typical changes of fundus photographs before and after treatment in the two groups are shown in Figure 2.
8. There was no significant difference of ETDRS vision score between two groups before treatment; however, the ETDRS vision score in the treatment group was significantly higher than that of the control group after six months' treatment (78 (72, 82) vs 72 (67, 80), *P* < 0.001) as shown in Table 6.
9. There were no significant differences of cube average thickness (CAT), central subfield thickness (CST), and cube volume (CV) in macular region between two groups before treatment as shown in Table 7.
10. As shown in Figure 3, the treatment group's CAT, CST, and CV were significantly lower after treatment (CAT: 286 (278, 302) vs 282 (270, 295) *μ*m, *P* < 0.0001; CST: 251 (239, 274) vs 248 (235, 265) *μ*m, *P* < 0.0001; CV: 10.3 (10.0, 10.9) vs 10.1 (9.7, 10.6) mm^3^, *P* < 0.0001), while there were no significant differences of control group's CAT, CST and CV before and after treatment (CAT: 287 (279, 294) vs 287 (279, 295) *μ*m, *P*=0.27; CST: 250 (240, 266) vs 252 (238, 266) *μ*m, *P*=0.72; CV: 10.4 (10.1, 10.6) vs 10.4 (10.1, 10.7) mm^3^, *P*=0.53). The typical changes of OCT images and parameters before and after treatment in the two groups are shown in Figure 4.
11. There were no significant differences of serum VEGF levels between two groups before treatment; however, the treatment group's VEGF level was significantly lower than that of the control group after six months' treatment (0.16 (0.10, 0.23) vs 0.23 (0.12, 0.64), *P*=0.02) as shown in Table 8. The serum VEGF levels of the treatment group were significantly lower after six months' treatment (0.21 (0.14, 0.58) vs 0.16 (0.10, 0.23) ng/ml, *P*=0.0026); however, there was no significant difference of the control group's VEGF levels before and after treatment (0.21 (0.13, 0.66) vs 0.23 (0.12, 0.64) ng/ml, *P*=0.85) as shown in Figure 3.
12. There were no significant differences of hypoglycemic drugs, hypotensive drugs, and other basic treatments between two groups during six months' treatment as shown in Table 9.

## 4. Discussion

As we know, Youyou Tu, the Nobel Prize winner, was inspired by the ancient medical document and discovered artemisinin by changing the drug extraction method. We could seek inspiration from traditional theories and experiences from the valuable resource of Chinese medicine. According to Chinese medicine literature, “the liver and kidney share the same origin and the eye and kidney share the same treatment.” The main functions of *Abelmoschus manihot* are “clearing heat and dampness, promoting blood circulation for removing obstruction in collaterals, and reducing swelling and detoxifying” [13]. Meanwhile, *Abelmoschus manihot* are effectively and commonly used for diabetic nephropathy [14–16]. These theories and experiences induced our hypothesis of applying *Abelmoschus manihot* for DR treatment. The pharmacological study showed that total flavone of *Abelmoschus* (TFA) is the major active component of *Abelmoschus manihot*. TFA is mainly composed of several flavonoids including quercetin, hyperoside, and isoquercitrin [17, 18]. It was shown that quercetin has a dose-dependent effect in inhibiting proliferation of rhesus macaque choroid-retinal endothelial cells [19] and reducing apoptosis of retinal ganglion cell layer and whole retinal vessels in diabetic rats [20, 21]. Meanwhile, the previous study has confirmed that quercetin could significantly inhibit the expression of retinal VEGF in diabetic rats [22]. High glucose induced the VEGF overexpression in the retina, which could damage the blood-retinal barrier (BRB) and result in pathogenesis of DR through multiple pathways. VEGF could accelerate the apoptosis of retinal pericytes (RPCs) and abnormal proliferation of retinal endothelial cells (RECs) [23, 24] and destroy intercellular tight-junctions proteins as occludin and claudin [25, 26]. It also could induce the increased expression of intercellular adhesion molecule-1 [27], inhibit the antagonistic effect of pigment epithelium-derived factor (PEDF) on VEGF [28], and promote the cell swallowing and endothelial cell perforation. Therefore, we aimed to explore the possible therapeutic effect of *Abelmoschus manihot* (Jiahua tablets) on treating NPDR in the present study.

The fundus photograph was comprehensively used in DR screening because of the convenient operation for physicians and easy cooperation for patients. But, there was disadvantage of unclear images in patients with severe cataract or small pupil [29]. The fundus fluorescein angiography (FFA) has no extra requirements for crystalline lens and pupils while the blood pressure, glucose, and liver and kidney functions need to be strictly controlled for using fluorescein sodium contrast agent. However, it was difficult to widely make angiography examinations in this study because the procedure of FFA was more time-consuming and expensive than fundus photograph [30]. In addition, the related study suggested that the sensitivity and accuracy of fundus photography are higher than of FFA combined with fundus photography (sensitivity: 0.63 vs 0.54; accuracy: 0.81 vs 0.79) [31]. The specificity of fundus photography is comparable with FFA combined with fundus photography (0.93 vs 0.97) in diagnosing polypoidal choroidal vasculopathy [31]. Meanwhile, in order to avoid the short-coming of fundus photography, NPDR patients with severe cataract of both eyes and small pupils with high intraocular pressure have been excluded before enrollment. Therefore, the fundus photography was chosen for the NPDR patients in our study. The study results suggested that the remission rate of NPDR severity in the treatment group was significantly higher than that of the control group (25.4% vs 9.3%, *P*=0.01) and the progression rate of NPDR severity in treatment group was significantly lower than that of the control group (4.2% vs 18.7%, *P*=0.007) after six months' treatment. It could be concluded that Jiahua tablets in the treatment group played an important role in improving the NPDR degrees because there were no significant differences of risk factors (blood glucose, blood lipid, blood pressure, age, and DM duration) and basic treatments (hypoglycemic, hypotensive, lipid regulation, and antiplatelet agents) between two groups during the study period. Additionally, the study results showed that the adverse reactions were not increased by Jiahua tablets because there were no significant differences of liver and kidney functions between two groups after six months' treatment. The efficacy and safety of *Abelmoschus manihot* in treating NPDR were preliminarily confirmed.

Vision, acting as the important indicator of DR improvement, was also compared between both groups before and after treatment. In this study, the ETDRS vision chart derived from the “Early Treatment Diabetic Retinopathy Study,” is recommended by the “National Eye Institute” and “Food and Drug Administration” of America. It is also considered as a recognized method for assessing visual acuity in clinical trials [32, 33]. It is more accurate to evaluate the levels of DR vision due to the objective quantification of severe vision by the ETDRS vision chart [34]. Our present results suggested that there were no significant differences of ETDRS scores between two groups before treatment (73 (67, 79) vs 74 (68, 80) points, *P*=0.26). However, the ETDRS score of the treatment group was significantly higher than that of the control group after six months' treatment (78 (72, 82) vs 72 (67, 80) points, *P* < 0.001). Similar to the above discussion about NPDR remission and progression rates, the improvement of NPDR vision in the treatment group was dependent neither of strict control of risk factors such as blood glucose, blood pressure, and blood lipids nor of the difference of basic treatments between two groups. The additional benefit of vision in the treatment group was mainly attributed to oral administration with *Abelmoschus manihot*.

DME is the most frequent cause of vision loss in DR. The severity of DME is usually evaluated by OCT which is a noncontact and noninvasive optical imaging technology. There was remarkable development of enhanced depth imaging (EDI) about OCT technology, which has improved the image quality of deeper structures. Swept-source OCT (SS-OCT) especially enables the choroid to be imaged at greater depth and using shorter acquisition times than those required for EDI using spectral-domain OCT (SD-OCT) [35, 36]. Both OCT devices had comparable repeatability for retinal thickness measurement in normal eyes and eyes with retinal disease [37]. The recent study suggested that diabetic choroidopathy (DC) should be evaluated in clinical trials of drugs targeting DR because vascular changes similar to those in DR are occurring in DC [38]. The preferred evaluation method of choroidal vascular density and volume in DC is SS-OCT because of particularly higher -resolution imaging of the choroid and deeper signal penetration by longer wavelength [39]. However, the relevant study indicated that there was no significant difference of choroidal thickness between NPDR alone and NPDR combined with DME. It also suggested that the notable submacular choroidal pathology might only become present in later stages of DR [39]. Besides, the OCT instrument of Cirrus HD 5000 used in this study is very suitable for clinical research due to the characteristics of convenient operation, stable performance, clear image, automatic request, and vertical comparison [40]. Meanwhile, there was an age-related normal Chinese database for comparison in the instrumental system of Cirrus HD 5000. Therefore, the SD-OCT instrument of Zeiss Cirrus HD 5000 was chosen for the NPDR patients in our study. The present study suggested that there were no significant differences of CAT, CST, and CV in the macular region between two groups before treatment; however, the treatment group's CAT, CST, and CV were significantly lower after six months' treatment (CAT: 286 (278, 302) vs 282 (270, 295) *μ*m, *P* < 0.0001; CST: 251 (239, 274) vs 248 (235, 265) *μ*m, *P* < 0.0001; CV: 10.3 (10.0, 10.9) vs 10.1 (9.7, 10.6) mm^3^, *P* < 0.0001), while there were no significant differences of control group's CAT, CST, and CV before and after treatment (CAT: 287 (279, 294) vs 287 (279, 295) *μ*m, *P*=0.27; CST: 250 (240, 266) vs 252 (238, 266) *μ*m, *P*=0.72; CV: 10.4 (10.1, 10.6) vs 10.4 (10.1, 10.7) mm^3^, *P*=0.53). It is concluded that *Abelmoschus manihot* plays an important role in inhibiting macular edema caused by diabetic retinopathy in this study, although severe DME scarcely occurred in NPDR patients. Our further studies about interfering PDR and severe DME with *Abelmoschus manihot* have been started.

Previous studies showed that the DR progression could be alleviated by inhibiting the activity of VEGF in the retina because the VEGF upregulation was a key factor leading to the BRB destruction and the pathogenesis of DR [41]. Anti-VEGF agent can prevent the development of DR through the mechanism that it specifically combines with VEGF to form an immune complex and loses the ability to bind to VEGF receptor [42, 43]. It is difficult to assay VEGF levels directly in the retina or vitreous body of DR patients. So, in order to investigate the possible effect of *Abelmoschus manihot* on VEGF, the serum VEGF levels were assayed by VEGF ELISA kit in this cohort. The data showed that there was no significant difference of serum VEGF levels between two groups before treatment (0.21 (0.14, 0.58) vs 0.21 (0.13, 0.66) ng/ml, *P*=1.00). There was no significant difference of VEGF levels in the control group before and after treatment (0.21 (0.13, 0.66) vs 0.23 (0.12, 0.64) ng/ml, *P*=0.85). However, the VEGF levels in the treatment group were significantly improved after six months' treatment (0.21 (0.14, 0.58) vs 0.16 (0.10, 0.23) ng/ml, *P*=0.0026). It could be concluded that the change of serum VEGF levels were associated with *Abelmoschus manihot*, and the downregulation of VEGF might play an important role in its therapeutic effect on NPDR. Our future work will design relevant experiments in vivo and in vitro to explore the molecular mechanisms underlying the therapeutic effect of *Abelmoschus manihot* on DR.

## 5. Conclusions

In summary, the present study offered the first clinical evidence that *Abelmoschus Manihot* could improve the severity of NPDR, ETDRS vision scores, macular edema, and serum VEGF levels. It could be taken as a novel complementary and alternative strategy for treating type 2 diabetic nonproliferative retinopathy.

## Figures and Tables

**Table 1:** Comparisons of demographic characteristics between two groups.

	T group (*n* = 40)	C group (*n* = 40)	*P*
Sex (male/female)	23/17	27/13	0.36
Age (years)	61 (52, 64)	55 (50, 63)	0.16
DM duration (years)	10 (5, 15)	8 (5, 13)	0.28
BMI (kg/m^2^)	25.9 ± 3.6	26.4 ± 3.3	0.48

T: treatment; C: control; BMI: body mass index.

**Table 2:** Comparisons of case terminations between two groups after treatment.

	T group (*n* = 38)	C group (*n* = 39)	*P*
Total terminated case	2 (5.0%)	1 (2.5%)	1.00
Causes of termination			
Automatic quit	1	1	1.00
Poor compliance	1	0	
Drug reaction	0	0	
Adverse events	0	0	

T: treatment; C: control.

**Table 3:** Comparisons of biochemical indicators and blood pressure between two groups before and after treatment.

	T group		C group		*P*_1_	*P*_2_
	Before (*n* = 40)	After (*n* = 38)	Before (*n* = 40)	After (*n* = 39)		
FBG (mmol/L)	8.7 ± 2.6	7.8 ± 1.9	8.2 ± 2.9	8.1 ± 2.2	0.37	0.47
PBG (mmol/L)	12.7 ± 2.2	10.5 ± 2.1	11.9 ± 3.0	10.8 ± 2.5	0.19	0.49
HbA1c (%)	8.8 ± 1.7	7.8 ± 1.8	9.1 ± 2.4	7.8 ± 1.4	0.51	0.95
TC (mmol/L)	4.3 ± 1.0	4.3 ± 1.0	4.1 ± 1.0	4.2 ± 1.0	0.41	0.53
TG (mmol/L)	1.7 (1.1, 2.4)	1.5 (1.2, 2.2)	1.6 (1.1, 2.5)	1.7 (1.1, 2.2)	0.77	0.74
HDL (mmol/L)	1.2 ± 0.3	1.4 ± 0.3	1.2 ± 0.3	1.3 ± 0.3	0.74	0.13
LDL (mmol/L)	2.7 ± 0.8	2.9 ± 0.9	2.5 ± 0.8	2.8 ± 1.0	0.48	0.70
ALT (U/L)	26 (18, 42)	29 (20, 38)	30 (19, 40)	29 (21, 35)	0.60	0.77
AST (U/L)	22 (17, 27)	22 (19, 28)	22 (18, 28)	23 (20, 29)	0.42	0.56
BUN (mmol/L)	6.7 ± 2.0	6.5 ± 1.5	6.2 ± 1.2	6.0 ± 1.3	0.22	0.14
Scr (umol/L)	67 (60, 81)	71 (61, 80)	68 (57, 75)	70 (58, 76)	0.61	0.50
eGFR (mL/min)	97.5 ± 25.7	98.3 ± 22.1	99.8 ± 17.2	98.8 ± 16.6	0.64	0.91
SBP (mmHg)	128 ± 12	129 ± 8	126 ± 10	130 ± 8	0.32	0.93
DBP (mmHg)	75 ± 10	77 ± 5	76 ± 8	78 ± 6	0.54	0.31

T: treatment; C: control; SBP: systolic blood pressure; DBP: diastolic blood pressure; P_1_: T group vs C group before treatment; P_2_: T group vs C group after treatment.

**Table 4:** Comparisons of NPDR severity between two groups before and after treatment.

	T group		C group		*P*_1_	*P*_2_
	Before (*n* = 75)	After (*n* = 71)	Before (*n* = 77)	After (*n* = 75)		
Mild	31 (41.3%)	42 (59.2%)	31 (40.2%)	27 (36.0%)	0.69	0.008^∗^
Moderate	31 (41.3%)	25 (35.2%)	36 (46.8%)	35 (46.7%)		
Severe	13 (17.4%)	4 (5.6%)	10 (13.0%)	13 (17.3%)		

T: treatment; C: control; P_1_: T group vs C group before treatment; P_2_: T group vs C group after treatment, ^∗^*P* ≤ 0.05.

**Table 5:** Comparisons of variations of NPDR severity between two groups after treatment.

	T group (*n* = 71)	C group (*n* = 75)	*P*
Improved	18 (25.4%)	7 (9.3%)	0.01^∗^
Maintained	50 (70.4%)	54 (72.0%)	0.83
Aggravated	3 (4.2%)	14 (18.7%)	0.007^∗^

T: treatment; C: control; ^∗^*P* ≤ 0.05.

**Table 6:** Comparisons of ETDRS vision score between two groups before and after treatment.

	T group		C group		*P*_1_	*P*_2_
	Before (*n* = 75)	After (*n* = 71)	Before (*n* = 77)	After (*n* = 75)		
ETDRS score	73 (67, 79)	78 (72, 82)	74 (68, 80)	72 (67, 80)	0.26	<0.001^∗^

T: treatment; C: control; P1: T group vs C group before treatment; P2: T group vs C group after treatment, ^∗^*P* ≤ 0.05.

**Table 7:** Comparisons of macular thicknesses between two groups before treatment.

	T group (*n* = 75)	C group (*n* = 77)	*P*
CAT (um)	286 (278, 302)	287 (279, 294)	0.67
CST (um)	251 (239, 274)	250 (240, 266)	0.78
CV (mm^3^)	10.3 (10.0, 10.9)	10.4 (10.1, 10.6)	0.62

T: treatment; C: control.

**Table 8:** Comparisons of serum VEGF levels between two groups before and after treatment.

	T group		C group		*P*_1_	*P*_2_
	Before (*n* = 40)	After (*n* = 38)	Before (*n* = 40)	After (*n* = 39)		
VEGF (ng/ml)	0.21 (0.14, 0.58)	0.16 (0.10, 0.23)	0.21 (0.13, 0.66)	0.23 (0.12, 0.64)	1.00	0.02^∗^

T: treatment; C: control; P_1_: T group vs C group before treatment; P_2_: T group vs C group after treatment, ^∗^*P* ≤ 0.05.

**Table 9:** Comparisons of hypoglycemic, hypotensive, and other basic treatment drugs between two groups.

	T group (*n* = 38)	C group (*n* = 39)	*P*
*Hypoglycemic drugs*			
Metformin	16	17	0.90
Sulfonylureas	16	12	0.30
Glinides	1	0	0.49
Acarbose	18	14	0.31
Thiazolidinedione	3	3	1.00
Insulin	2	0	0.24
Insulin-analog	21	23	0.74
GLP-1	4	6	0.77
DPP-4	12	19	0.13
SGLT-2	3	1	0.59
*Hypotensive drugs*			
CCB	17	11	0.13
*β*RB	7	9	0.61
ACEI	2	1	0.98
ARB	8	8	0.95
Diuretic	3	4	1.00
*Other treatments*			
Aspirin	7	10	0.45
Statins	13	19	0.20
ARI	1	0	0.49

T: treatment; C: control; CCB: calcium channel blockers; *β*RB: *β* receptor blockers; ACEI: angiotensin-converting enzyme inhibitors; ARB: angiotensin II receptor blockers; ARI: aldose reductase inhibitor.

## Data Availability

The data used to support the findings of the study are available from the corresponding author upon request.
